# Fasting potentiates the anticancer activity of tyrosine kinase inhibitors by strengthening MAPK signaling inhibition

**DOI:** 10.18632/oncotarget.3689

**Published:** 2015-03-18

**Authors:** Irene Caffa, Vito D'Agostino, Patrizia Damonte, Debora Soncini, Michele Cea, Fiammetta Monacelli, Patrizio Odetti, Alberto Ballestrero, Alessandro Provenzani, Valter D. Longo, Alessio Nencioni

**Affiliations:** ^1^ Department of Internal Medicine, University of Genoa, Genoa, Italy; ^2^ Laboratory of Genomic Screening, Centre for Integrative Biology, CIBIO, University of Trento, Trento, Italy; ^3^ IRCCS AOU San Martino-IST, Istituto Nazionale per la Ricerca sul Cancro, Genoa, Italy; ^4^ Longevity Institute, School of Gerontology, Department of Biological Sciences, University of Southern California, Los Angeles, CA, USA; ^5^ IFOM, FIRC Institute of Molecular Oncology, Milan, Italy

**Keywords:** tyrosine kinase inhibitors, fasting, MAPK pathway, E2F transcription factors, cell cycle regulation

## Abstract

Tyrosine kinase inhibitors (TKIs) are now the mainstay of treatment in many types of cancer. However, their benefit is frequently short-lived, mandating the search for safe potentiation strategies. Cycles of fasting enhance the activity of chemo-radiotherapy in preclinical cancer models and dietary approaches based on fasting are currently explored in clinical trials. Whether combining fasting with TKIs is going to be potentially beneficial remains unknown. Here we report that starvation conditions increase the ability of commonly administered TKIs, including erlotinib, gefitinib, lapatinib, crizotinib and regorafenib, to block cancer cell growth, to inhibit the mitogen-activated protein kinase (MAPK) signaling pathway and to strengthen E2F-dependent transcription inhibition. In cancer xenografts models, both TKIs and cycles of fasting slowed tumor growth, but, when combined, these interventions were significantly more effective than either type of treatment alone. In conclusion, cycles of fasting or of specifically designed fasting-mimicking diets should be evaluated in clinical studies as a means to potentiate the activity of TKIs in clinical use.

## INTRODUCTION

Tyrosine kinase inhibitors (TKIs) are now the mainstay of treatment in several types of cancer. By interfering with the tyrosine kinase activity of mutated or overexpressed oncogenes, such as Epidermal Growth Factor Receptor (EGFR) or Human Epidermal Growth Factor Receptor 2 (HER2), or by simultaneously blocking multiple signaling pathways that are relevant to cancer (or to cancer angiogenesis), these drugs target biological functions that cancer cells are critically reliant on, ultimately causing cancer cell death and tumor shrinkage [1]. EGFR TKIs have a proven superiority over standard chemotherapy in non-small-cell lung cancer (NSCLC) with mutated EGFR and Anaplastic Lymphoma Kinase (ALK) TKIs are successfully employed in NSCLC with translocated ALK [2]. HER2 TKIs, such as lapatinib, are used in breast cancer with amplified HER2 [3] while Vascular Endothelial Growth Factor Receptor (VEGFR) TKIs and multitarget TKIs are commonly employed in renal cell carcinoma, hepatocellular carcinoma, thyroid cancer, gastrointestinal stromal tumors (GIST), and in previously treated colorectal cancer [4-7]. In sensitive tumors, disease remissions can frequently be achieved with these agents. However, patients will sooner or later face relapses due to the emergence of resistant cancer cell clones and eventually succumb to their disease [2-4, 8]. Thus, there remains a critical need for strategies to safely increase the effectiveness of TKIs [8].

Recent discoveries indicate that an important Achilles' heel of many types of cancer cells is their inability to adapt to starvation [9, 10]. While healthy cells respond to nutrient and growth factor deprivation by activating maintenance and stress response mechanisms that make them more resistant to different types of insults, including chemotherapy, this type of response is frequently compromised in cancer cells, primarily as a consequence of aberrant oncogene activation [9, 10]. Instead of reducing the activity of growth promoting signalling pathways and protein synthesis, starved cancer cells may boost both processes, ultimately facing metabolic imbalance and becoming prone to oxidative stress, caspase activation, DNA damage, and apoptosis [9]. In pre-clinical models, cycles of fasting were found to be per se sufficient to slow tumor growth, matching in some cases the efficacy of chemotherapy, and to synergize with chemotherapeutics and radiotherapy when applied in combination with them [9, 11, 12]. Another possible advantage of administering chemotherapy during fasting is that its overall tolerability appears to be increased, potentially allowing to administer higher doses of chemotherapeutics without severe toxicity [10, 13, 14].

Several clinical trials are currently studying the effects of fasting or of fasting-mimicking diets in patients undergoing chemotherapy (NCT01304251, NCT01175837, NCT00936364, NCT01175837, NCT01802346, NCT02126449). Preliminary clinical observations indicated that this type of dietary interventions are feasible and can be safely introduced [14]. In 2009, Safdie and colleagues reported on a series of ten patients diagnosed with a variety of malignancies who voluntarily fasted prior to (48-140 h) and/or following (5-56 h) chemotherapy (an average of 4 cycles of various chemotherapy drugs in combination with fasting) [14]. More recently, evidence of potential beneficial effects of fasting in patients receiving chemotherapy in terms of reduced risk of leukopenia has been reported [13]. In the light of these results, further evaluations of the potential of these approaches, both at the preclinical and at the clinical level, are warranted [15, 16].

The purpose of this preclinical study was to determine whether administering TKIs during fasting would increase their efficacy and to investigate the mechanisms underlying these effects.

## RESULTS

### Starvation conditions potentiate the anti-proliferative effects of TKIs in cultured cancer cells

**Figure 1:**
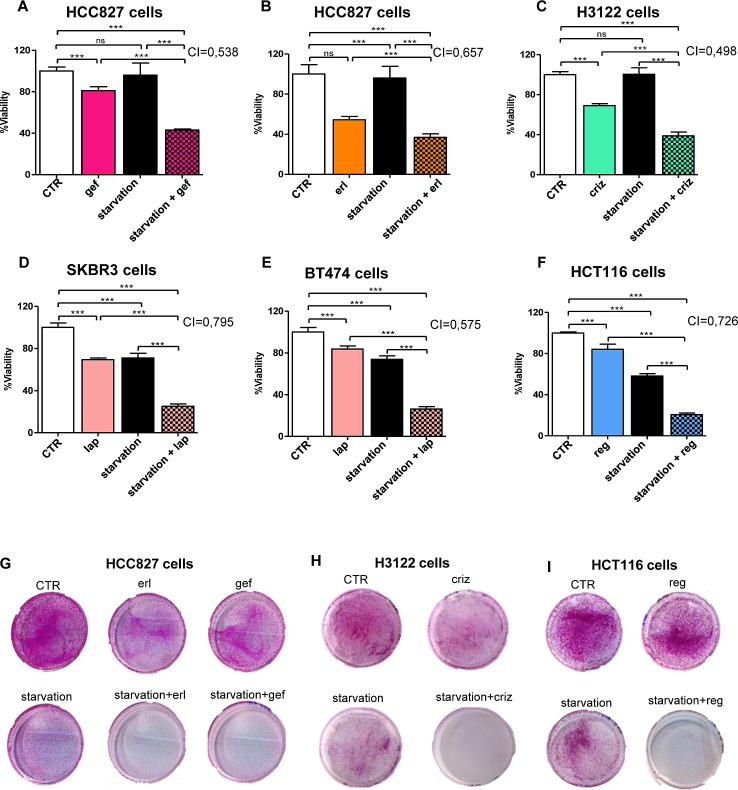
Starvation conditions synergistically increase the antiproliferative effects of TKIs in cultured cancer cells HCC827 (EGFR mutated, exon 19 EGFR deletion), H3122 (ALK+ NSCLC), SKBR3, BT474 (HER2+ breast cancer), or HCT116 (colorectal cancer) cells were plated in 96 well plates in regular culture medium. 24 h later, the cell medium was removed and cells were incubated either in regular medium (CTR) or in starvation medium (starvation). 24 h later, 10 nM gefitinib (gef), 100 nM erlotinib (erl), 400 nM crizotinib (criz), 100 nM lapatinib (lap), or 300 nM regorafenib (reg) were added where indicated. 72 h later, viability was detected by CellTiter96 Aqueous1. CIs for the combinations TKI-starvation are indicated within each panel. G-I, 4×10^5^ HCC827, 1×10^5^ H3122, 5×10^4^ HCT116 cells/dish were plated in 10 cm dishes and treated with erlotinib, gefitinib, crizotinib, or regorafenib as in A-F. 5 days later, cell medium was removed and cells were cultured for two additional days in regular culture medium. Thereafter, cells were fixed, stained with SRB and imaged.

Experiments were conducted to ascertain whether culture conditions mimicking the metabolic consequences of fasting (i.e. low glucose - 0.5 g/l - and low serum -1% FBS) would potentiate the growth-inhibiting activity of TKIs in current clinical use, including the EGFR TKIs erlotinib (Tarceva) and gefitinib (Iressa), the ALK TKIs crizotinib (Xalkori) and TAE684, the HER2/EGFR TKIs lapatinib (Tykerb) and CP724714, and the multitarget TKI regorafenib (Stivarga) (Figure 1 and Supplementary Figure 1). These compounds were tested in appropriate cellular models, which included the NSCLC cells with mutated EGFR, HCC827, the NSCLC cells carrying the EML4-ALK translocation, H3122, the breast cancer cells with amplified HER2, BT474 and SKBR3, and the regorafenib-sensitive colorectal cancer cells HCT116, by measuring viability with the MTS-based assay CellTiter96 Aqueous 1. After a four-day treatment, starvation conditions were sufficient to slow cancer cell growth in SKBR3, BT474, and HCT116 cells (Figure 1D-F), but not in HCC827 and H3122 cells (Figure 1A-C), although extending the starvation period from four to six days visibly affected cell growth in the latter two cell lines, too (Figure 1G, H). In all of the cellular models, a synergistic potentiation of the TKI activity by starvation was readily evident and was documented by the calculation of CIs (Figure 1A-I, Supplementary Figure 1A, B). Measuring cell viability with an alternative assay, the sulforhodamine B colorimetric assay [17], yielded similar results and readily revealed a cooperation between starvation conditions and TKIs, too (data not shown).

Notably, the potentiation of TKI activity was apparently not at the cost of a loss in specificity, since starved breast cancer cells without HER2 amplification (MDA-MB-231 cells) were still insensitive to lapatinib and NSCLC without mutated EGFR or translocated ALK (A549 cells) remained insensitive to erlotinib and crizotinib despite starvation (Supplementary Figure 1C-E).

### Evidence for a key role of MAPK signaling inhibition in the potentiation of TKI activity by starvation

**Figure 2:**
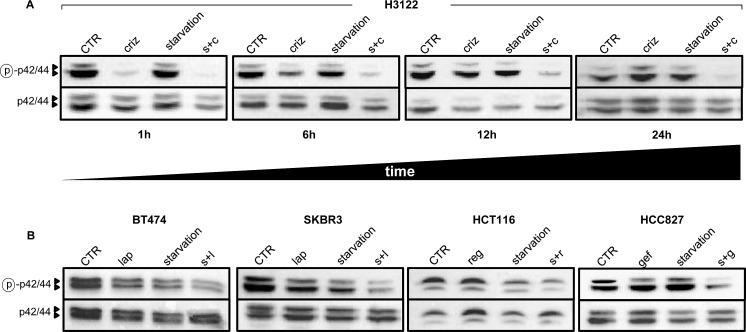
Starvation conditions enhance TKI-mediated inhibition of MAPK signaling 6 × 10^5^ H3122 cells were plated in 100 mm Petri dishes in regular medium. 24 h later, the cell medium was removed and cells were incubated either in regular medium (CTR) or in starvation medium. 24 h later cells were treated or not with 400 nM crizotinib (criz) for the indicated amounts of time. Finally, cell lysates were prepared and phospho p42/44 (ERK, Thr202/Tyr204) and total p42/p44 were detected by immunoblotting. B, 9 × 10^5^ BT474, 8 × 10^5^ SKBR3, 5 × 10^5^ HCT116, and 5 × 10^5^ HCC827 cells/dish were plated in 100 mm Petri dishes, pre-starved (or not) as in A, and finally stimulated for 24 with 100 nM lapatinib (lap), 300 nM regorafenib (reg) or 10 nM gefitinib (gef). Finally, cell lysates were prepared and phospho-p42/44 (ERK, Thr202/Tyr204) and total p42/p44 were detected by immunoblotting. s+c: starvation+crizotinib; s+l: starvation+lapatinib; s+r: starvation+regorafenib; s.g: starvation+gefitinib.

In cancer cells with mutated receptor tyrosine kinases (RTKs) or oncogenes with kinase activity, growth is typically driven by the constitutive activation of downstream signaling cascades, such as the mitogen-activated protein kinase (MAPK) pathway [18]. Inhibition of these pathways by TKIs is typically rapid, but frequently short-lived, given the activation of compensatory feedback mechanisms which limit the efficacy of the TKIs themselves [19]. We monitored the phosphorylation status of ERK as a reading frame for the activity of the MAPK signaling cascade in cells treated with TKIs with or without starvation. Experiments performed with crizotinib and lapatinib demonstrated a striking reduction of phospho-ERK after a 1-h treatment [Figure 2A and [20]]. However, prolonged exposure to the TKI would typically result in a recovery in ERK phosphorylation, which returned to baseline levels, or even topped them, by 24 h of exposure in all of the cell lines tested (Figure 2A-B). Starvation caused a reduction in ERK phosphorylation in some cell line (BT474, SKBR3, and HCT116), but not in H3122 and HCC827. However, in every instance, the combination of starvation plus TKI was the most effective at maintaining ERK phosphorylation inhibition in cancer cells (Figure 2A-B). Therefore, these findings are in line with those from the viability assays and suggest that the cooperation between TKIs and starvation conditions may rely on the ability of the latter to prevent compensatory or alternative mechanisms that can bypass signaling cascade inhibition by TKIs.

**Figure 3:**
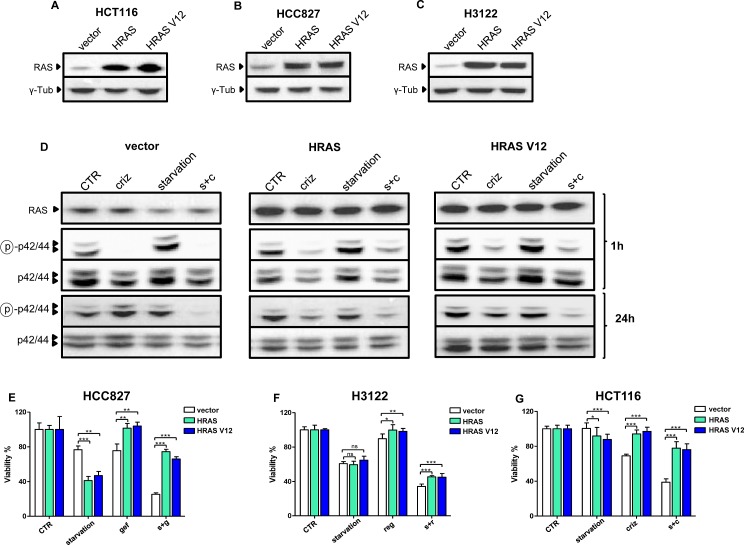
Overexpressed HRAS and HRAS V12 prohibit TKI activity potentiation through starvation A-C, HCT116, HCC827 and H3122 cells were retrovirally engineered to overexpress wild type HRAS, HRAS V12, or the control vector PBP. Successfully infected cells were selected with puromycin and used for protein lysate generation. HRAS and γ-tubulin levels were detected by immunoblotting. D, 6 × 10^5^ PBP, HRAS and HRAS V12 H3122 cells/dish were plated in 100 mm Petri dishes in regular medium. 24 h later, the cell medium was removed and cells were incubated either in the same medium (CTR) or in starvation medium. 24 h later cells were treated or not with 400 nM crizotinib (criz). 1 h later or 24 h later cell lysates were prepared and phospho p42/44 (ERK, Thr202/Tyr204), total p42/p44, and HRAS (as detected in lysates generated after a 1 h incubation) were revealed by immunoblotting. E-G, 2 × 10^3^ PBP, HRAS, or HRAS V12 H3122, HCT116, and HCC827 cells/well were plated in 96 well plates in regular culture medium. 24 h later, the cell medium was removed and cells were incubated either in regular medium (CTR) or in starvation medium (starvation). 24 h later, 10 nM gefitinib (gef), 400 nM crizotinib (criz) or 300 nM regorafenib (reg) were added where indicated. 72 h later, cell viability was detected by CellTiter96 Aqueous1. s+g: starvation+gefitinib; s+r: starvation+regorafenib; s+c: starvation+crizotinib.

We subsequently, reasoned that, if this were the case, a constitutive activation of the MAPK signaling pathway would prohibit or, at least, reduce, the observed potentiation of TKI activity by starvation. Thus, we engineered HCC827, HCT116, and H3122 cells to overexpress either a wild type HRAS allele or HRAS V12 (Figure 3A-C) and treated these cells with gefitinib, regorafenib, or crizotinib with or without starvation, respectively. As shown for H3122 cells, expressing HRAS or HRAS V12 indeed led to higher levels of phosphorylated ERK in response to crizotinib alone or to crizotinib coupled to starvation conditions (Figure 3D). Interestingly, we found that the expression HRAS alleles sensitized two out of three cancer cell lines (H3122 and HCC827) to starvation, while HCT116 cells were not sensitized, possibly because this cell line already harbors a mutated KRAS, which may make these cells less affected by the genetic manipulation that we performed (Figure 3E-G). However, in all of the cell lines, both HRAS and HRAS V12 blunted the anti-proliferative activity of the TKIs, both alone and in combination with starvation, indicating that inhibition of the MAPK pathway indeed plays a key role in mediating the antitumor effects of TKIs in both conditions (Figure 3E-G).

### Starvation enhances TKI-mediated inhibition of MAPK-driven transcription

To gain further insights into the mechanisms underlying the potentiation of TKI activity by starvation we chose H3122 cells and compared the effects of crizotinib, starvation and their combination in terms of global gene expression as detected by standard gene expression microarrays. Six different comparisons were performed (starvation vs. control, crizotinib vs. control, starvation vs. crizotinib, crizotinib plus starvation vs. control, crizotinib plus starvation vs. starvation, and crizotinib plus starvation vs. crizotinib) and the differentially expressed genes (DEGs) were quantified as summarized in Supplementary Table 1. Here, it is noteworthy that, while all of the treatments (or their combinations) changed gene expression (vs. control cells) by over a thousand genes (2,308 DEGs for starvation; 1,305 DEGs for crizotinib; 2,676 DEGs for crizotinib plus starvation) comparing starvation vs. crizotinib yielded a much lower number of DEGs (568), suggesting that the two types of treatment have similar biological effects in cancer cells. In line with this notion, according to a GO analysis, cells treated with crizotinib and starved cells shared most of the top, common GO categories (Table 1 and Supplementary Tables 2 and 3) while only two GO categories appeared to be unique of starved cells and of cells exposed to crizotinib. Specifically, it emerged that, in starved cells, down-regulated DEGs clustered in categories related to cell cycle control and DNA damage response (Table 1 and Supplementary Table 2), while up-regulated DEGs were only loosely convergent in categories related to endoplasmic reticulum functionalities. The same analysis conducted in crizotinib-treated cells gave similar results, being the top categories of down-regulated DEGs the same as in the previous analysis (Table 1 and Supplementary Table 2), while, again, up-regulated DEGs were not convergent in any ontological category. Notably, when considering only the down-regulated DEGs that were present in the top four affected canonical pathways, the overlap between starvation and crizotinib was almost complete, with 48 out of 53 genes represented in the top three pathways modulated by starvation (vs. control) being also present in the top four pathways modulated by crizotinib (vs. control) (Supplementary Table 3). Therefore these analyses indicated that starvation and treatment with crizotinib share core transcriptional effects.

**Table 1:** Gene Ontology Category analysis of DEGs in H3122 cells in response to crizotinib, starvation, or their combination

Starvation and crizotinib (Common GO Category)		
GO Category	Starvation p-value	Crizotinib p-value
Cell Cycle Control of Chromosomal Replication	6.31E-13	1.00E-13
Mitotic Roles of Polo-Like Kinase	1.86E-10	3.98E-14
Role of BRCA1 in DNA Damage Response	4.79E-09	3.16E-12
Role of CHK Proteins in Cell Cycle Checkpoint Control	1.17E-08	1.26E-11
Hereditary Breast Cancer Signaling	1.82E-07	6.03E-10
Mismatch Repair in Eukaryotes	1.91E-07	3.80E-08
ATM Signaling	3.63E-07	9.12E-08
Aryl Hydrocarbon Receptor Signaling	5.25E-07	2.34E-04
Cell Cycle: G2/M DNA Damage Checkpoint Regulation	1.62E-06	1.00E-10
Estrogen-mediated S-phase Entry	7.59E-06	5.89E-08
GADD45 Signaling	1.78E-05	5.75E-05
Cyclins and Cell Cycle Regulation	4.07E-05	5.25E-06
**Starvation only**		
Role of IL-17A in Psoriasis	1.05E-05	n.s.
Pyrimidine Deoxyribonucleotides De Novo Biosynthesis I	6.31E-04	n.s.
**Crizotinib only**		
DNA damage-induced 14-3-3σ Signaling	n.s.	5.75E-05
Remodeling of Epithelial Adherens Junctions	n.s.	2.34E-04

6 × 10^5^ H3122 cells/dish were plated in regular medium in 100 mm Petri dishes. 24 h later, the cell medium was removed and cells were incubated either in regular medium (CTR) or in starvation medium. 24 h later cells were treated or not with 400 nM crizotinib for additional 24 h before total RNA was isolated and utilized in standard gene expression microarrays.

**Figure 4:**
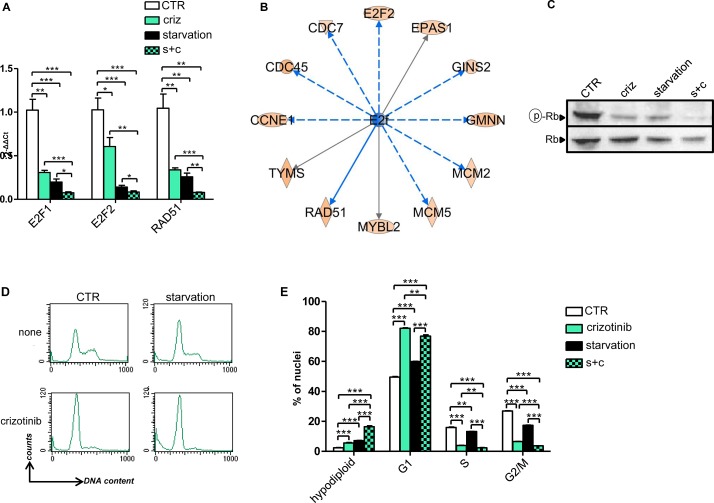
Gene expression and cell cycle regulation by starvation, crizotinib and their combination in H3122 cells A, 6 × 10^5^ H3122 cells were plated in 10 cm Petri dishes in regular medium. 24 h later, the cell medium was removed and cells were incubated for 24h either in regular medium (CTR) or in starvation medium. 24 h later cells were treated or not with 400 nM crizotinib for 24 h. Finally, total RNA was isolated and utilized for E2F1, E2F2 and RAD51 mRNA quantification by QPCR. B, Identification of upstream regulators of the en masse gene expression regulation by combined crizotinib plus starvation vs. crizotinib alone by IPA. C-E H3122 cells were treated as in A and utilized for either protein lysate generation or cell nuclei isolation and propidium iodide staining. C, phospho-RB (Ser807/811) and total RB were detected by immunoblotting. D, E, cell cycle was analyzed on a FACS Calibur by acquiring 10.000 events/sample.

In order to determine what starvation would add to the TKI in terms of gene expression regulation, we compared the arrays corresponding to crizotinib alone to those corresponding to crizotinib plus starvation. Also in this case, the number of DEGs (264) was found to be very limited, which is in line with the notion that both types of treatment likely act on the same genetic and molecular mechanisms. We reasoned that these 264 DEGs could represent a core of genes that may explain the stronger anti-proliferative effect exerted by combined crizotinib and starvation compared to crizotinib alone. Using functional enrichment we observed that the DEGs of cells treated with crizotinib and starvation clustered in canonical pathways within the area of cell cycle control and DNA damage (Supplementary Table 4). Among these genes, it is worth to notice the presence of important cancer genes such as E2F1, E2F2, and RAD51, which combined starvation and crizotinib down-regulated to a higher extent compared to these treatments alone (Supplementary Table 4). Notably, the effect of crizotinib, starvation and their combination on E2F1, E2F2, and RAD51 could be readily verified by QPCR (Figure 4A).

In the attempt to identify upstream regulators that could be responsible of the en masse gene co-regulation exerted by crizotinib plus starvation (vs. crizotinib alone), the family group of E2f factors (E2F1-6) was found as the most likely key proteins causing the observed changes in gene expression (Figure 4B and Supplementary Table 5). This prediction is based on the fact that many of the genes known to be experimentally modified by these proteins and transcription factors were overrepresented in our gene lists in a statistically significant fashion. Specifically, according to this type of analysis, gene expression modulation by combined crizotinib and starvation (vs. crizotinib) could be attributed to the activation of E2F6, a dominant negative inhibitor of other E2F family members [21], and of RB1, and to inhibition of the cell cycle promoting factors E2F1 and E2F4 [22]. Consistent with these findings, a reduction in phosphorylated RB that was more pronounced in cells treated with the combination crizotinib plus starvation as compared to crizotinib or starvation alone could be detected in H3122 by immunoblotting (Figure 4C). Finally, in cell cycle experiments, we found that, while crizotinib, starvation, and their combination all caused an accumulation of cells in the G1/0 phase of the cell cycle, the reduction in the cells proceeding to S and G2/M phase was more pronounced when the two treatments were given together. Moreover, combining crizotinib with starvation increased the rate of hypodiploid cell nuclei, which is consistent with an increase in cell death (Figure 4D, E). Therefore, taken together, these findings are consistent with the observation that inhibition of the MAPK signaling pathway by TKIs is made stronger and more durable by starvation (Figure 2) and that such inhibition plays a key role in the activity of combined TKI plus starvation (Figure 3).

### Fasting potentiates TKI activity *in vivo*

**Figure 5:**
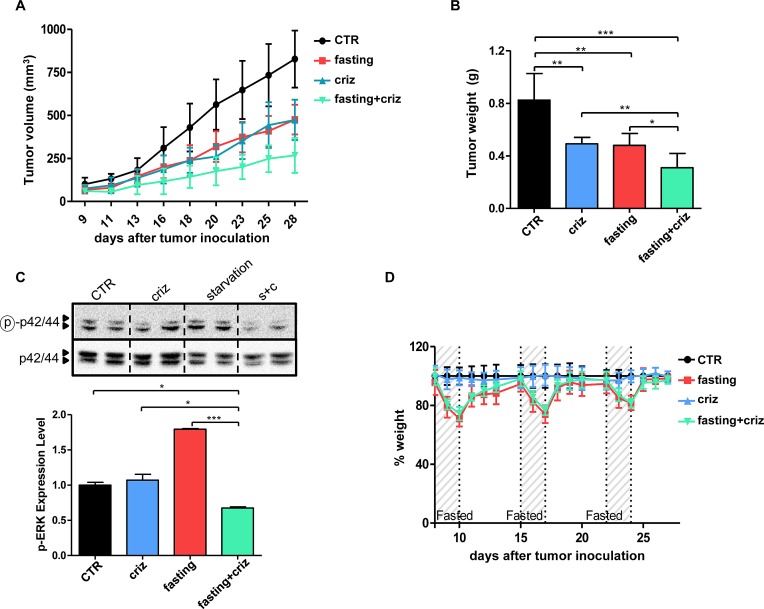
Fasting potentiates the anticancer activity of crizotinib *in vivo* A-D, Six- to eight-week-old BALB/c athymic mice (nu^+^/nu^+^) were injected s.c. with 5 × 10^6^ H3122 cells. When tumors become palpable, mouse were randomly assigned to one of four arms (six mice per treatment arm): control, normal diet; crizotinib (normal diet with crizotinib); fasting; fasting+crizotinib. After three weeks from the beginning of treatment, mice were euthanized and tumor masses were excised and weighted (B), before being used for protein lysate generation. Mouse weight (D) and tumor volume (A) were monitored daily (A, at d+21: CTR vs. crizotinib: p<0.01; CTR vs. fasting: p<0.01; crizotinib vs. crizotinib+fasting: p<0.01; fasting vs. crizotinib+fasting: p<0.01). C, Phospho-ERK (Thr202/Tyr204) and total ERK levels in protein lysates from tumor masses were monitored by immunoblotting. Band intensity quantification is presented in C, lower inset. *: p<0.05; **: p<0.01; ***: p<0.001.

**Figure 6:**
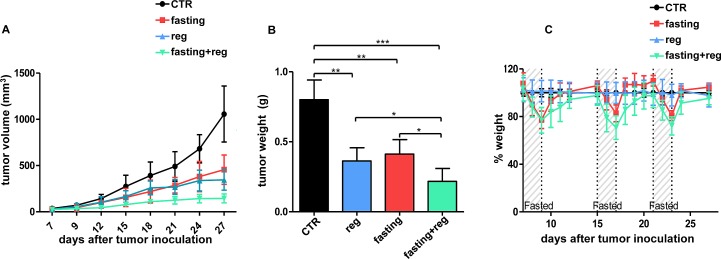
Fasting potentiates regorafenib *in vivo* activity in HCT116 xenografts A-C, Six- to eight-week-old BALB/c athymic mice (nu^+^/nu^+^) were injected s.c. with 2 × 10^6^ HCT116 cells. When tumors become palpable, mouse were randomly assigned to one of four arms (six mice per treatment arm): control, normal diet; regorafenib (normal diet with regorafenib); fasting; fasting+regorafenib. Mouse weight (C) and tumor size (A) were monitored daily (A, at day +21: CTR vs. regorafenib: p<0.01; CTR vs. fasting: p<0.01; regorafenib vs. regorafenib +fasting: p<0.05; fasting vs. regorafenib+fasting: p<0.01). After three weeks from the beginning of treatment, mice were euthanized and tumor masses were excised and weighted (B). *: p<0.05; **: p<0.01; ***: p<0.001.

Based on these premises, we sought for the proof-of-concept that starvation would increase the efficacy of TKIs also *in vivo*, using, to this end, well-established xenografts cancer models. In H3122 xenografts, both cycles of fasting and crizotinib effectively reduced tumor growth with no difference in terms of efficacy between the two approaches (Figure 5 A, B). However, the combination TKI+fasting was more effective than either type of treatment alone (Figure 5A, B). Notably, as observed *in vitro*, the combination of fasting with crizotinib was the only intervention to result in a significant reduction in phospho-ERK levels, whereas fasting alone was found to increase phospho-ERK *in vivo*, too (Figure 5C). Notably, although fasted mice did exhibit transient weight losses, they fully recovered their weight between one cycle and the next one (Figure 5D). Similar results were obtained with regorafenib in xenografts of the colorectal cancer cell line, HCT116 (Figure 6). After a three-week treatment, both regorafenib and cycles of fasting achieved a substantial reduction in tumor growth compared to the controls (Figure 6A, B). However, again, combined fasting and regorafenib were significantly more active than either type of treatment alone. Finally, also in this animal model, the weight lost by fasted animals was fully recovered between one treatment cycle and the following one (Figure 6C).

## DISCUSSION

In this study, we show for the first time that TKIs that are commonly administered for treating solid tumors become potentiated in their activity by starvation. We also demonstrate that starvation increases the efficacy with which TKI block cell signaling via the MAPK cascade and consequently affect gene expression. These results are of special relevance since they suggest possible new applications for short-term fasting or for specifically designed fasting-mimicking dietary regimens in combination with TKIs in oncology, which could replace much more toxic and less effective chemotherapy treatments.

Notably, compared to standard chemotherapy, TKIs typically have a lower toxicity and their administration rarely leads to gastrointestinal side effects with consequently reduced food intake and weight loss [1-4]. Thus, patients that are on chronic treatment with TKIs appear to be particularly well-suited for undergoing cycles of fasting (or of fasting-mimicking diets). In addition, because fasting was shown in earlier studies to promote cell cycle inhibition and to activate powerful self-protection mechanisms in normal cells [10], its use in combination with TKIs has the potential to reduce even further the toxicity of these inhibitors while broadening their range of activity. Therefore, coupling cycles of starvation with TKIs may be more feasible in a clinical context and allow a much better control of the disease.

The mechanism underlying the synergy between starvation, on the one hand, and TKIs, on the other, appears to be, at least in part, an inhibition of the signaling cascades controlled by aberrantly activated oncogenes (i.e. MAPK signaling), which is much more pronounced when the two interventions are combined than when they are administered separately. In line with this notion are the results of our microarray studies, which showed a strong overlap between the effects of starvation and crizotinib in terms of changes induced in the gene expression profile, and a synergy between the two interventions for the inhibition of E2F transcription factor-controlled transcription [22]. Formally, the demonstration of a key role for the inhibition of MAPK signaling in the activity of TKIs and of TKIs combined with starvation was obtained in cell lines engineered with wild type or oncogenic HRAS, since both HRAS alleles were able to partially reverse the effect of the TKI (alone and in combination with starvation) on cancer cell viability. Notably, it is possible that other mechanisms in addition to MAPK signaling inhibition also contribute to the synergistic potentiation of TKI activity by starvation, both *in vitro* and *in vivo*. Addressing this possibility may increase our understanding of this effect and also allow a better selection of the patients who are going to benefit the most from this combination.

Incidentally, of interest was also the observation that, in H3122 and HCC827 cells overexpressing HRAS alleles, starvation alone inhibited cell growth to a higher extent as compared to control cells. These findings suggest that, even if resistant to TKIs (administered in combination with starvation or not), cancer cell clones overexpressing HRAS are likely to be strongly affected by fasting (or fasting-mimicking diets), which could potentially avoid the expansion of TKI-resistant, HRAS overexpressing cells [20] or, at least, delay such an occurrence.

Dietary interventions that are less drastic than fasting, such as calorie restriction or protein restriction have previously been reported to achieve some of the benefits of fasting itself, namely, a partial protection from the toxicity of chemotherapy [23]. Whether they are also sufficient to achieve anticancer effects on established tumors or to potentiate the activity of anticancer agents is debated [23-26]. Addressing the effects of calorie or protein restricted regimens in combination with TKIs is going to be of extreme importance in the definition of the optimal diet that, when combined with these agents, will maximize their effectiveness [27].

In conclusion, our study shows that administering TKIs under starvation conditions achieves optimal anticancer effects by providing a full and durable inhibition of the MAPK cascade and, consequently, of E2F-mediated transcription. This work highlights a possible new application for fasting (or for specifically designed fasting-mimicking diets) in oncology in combination with TKIs which has the potential to reduce toxicity to the host while enhancing the efficacy of the therapy. Further validation of these preclinical observations in clinical studies should be considered.

## METHODS

### Cell lines and reagents

SKBR3, BT474, HCT116, HCC827 and Phoenix cells were purchased from ATCC (LGC Standards S.r.l., Milan, Italy). H3122 cells were a kind gift of Dr. Sacha Rothschild (University Hospital of Basel, Switzerland). Cells were passaged for less than 6 months before resuscitation for this study. These cell lines were authenticated at ATCC by analysis of eight short tandem repeat loci (CSF1PO, D13S317, D16S539, D5S818, D7S820, TH01, TPOX, and vWA) and of the Amelogenin gene (see the provider's web page). Cells were maintained in RPMI1640 medium supplemented with 10% FBS, penicillin (50 units/ml), and streptomycin (50 μg/ml) (LifeTechnologies, Italy). Regorafenib, crizotinib, erlotinib and gefitinib were purchased from Selleck Chemicals while lapatinib was a kind gift of GlaxoSmithKline. Puromycin and the protease/phosphatase inhibitor cocktail were purchased from Sigma Aldrich S.r.l. (Milan, Italy).

### Viability assay

4×10^3^ BT474, 3×10^3^ SKBR3, 1.7×10^3^ H3122, 1.5×10^3^ HCT116 or 2.5×10^3^ HCC827 cells/well were plated in 96 well plates in regular medium containing 10% FBS. 24 h later, the cell medium was removed, cells were washed twice with PBS and then incubated either in the same medium or in starvation medium (1% FBS, 0.5 g/l glucose and antibiotics). 24 h later cells were stimulated (or not) with lapatinib, CP724714, crizotinib, regorafenib, gefitinib, or erlotinib. Viability was determined 72 h later by CellTiter96 Aqueous1 (Promega) according to the manufacturer's instructions.

### Sulforodhamine B staining

4×10^5^ BT474, 3×10^5^ SKBR3, 1×10^5^ H3122, 5×10^4^ HCT116, or 2 x10^5^ HCC827 cells were plated in 60 mm Petri dishes in regular medium. 24 h later, the cell medium was removed, cells were washed twice with PBS and were incubated either in regular medium or in starvation medium. 24 h later cells were treated or not with lapatinib, crizotinib, regorafenib, gefitinib, or erlotinib. 5 days later, cell medium was removed and cells were cultured for two additional days in regular culture medium. Thereafter, the culture plates were fixed with cold 3% trichloroacetic acid at 4°C for 30 minutes, washed with cold water and dried overnight. Finally, the plates were stained with 0.4% sulforodhamine B (SRB) in 1% acetic acid, washed four times with 1% acetic acid to remove unbound dye, dried overnight and photographed.

### Cell cycle analysis

For cell cycle analysis, adhering cells were resuspended in a buffer containing 0.1% Na citrate, 0.1% Triton-X, and 50 μg/ml propodium iodide. Thereafter, the isolated cell nuclei were analyzed by flow cytometry using a FACS Calibur (Becton Dickinson, Milan, Italy).

### Retroviral transduction

pBABE-puro (PBP), PBP-HAS and PBP-HRAS V12 were purchased from Addgene (Cambridge, MA, USA). For retroviral transduction, 1 × 10^6^ Phoenix cells were plated in 60 mm Petri dishes and allowed to adhere for 24 h. Thereafter, cells were transfected with 4 μg plasmid DNA using TransIT-293 (Mirus Bio, Madison, WI) according to the manufacturer's instructions. Viral supernatants were harvested after 36, 48, 60 and 72h and used to infect H3122 cells (3 × 10^5^), HCT116 (2.5 × 10^5^) cells and HCC827 cells (3.5 × 10^5^) in 100 mm Petri dishes in the presence of 5 μg/ml protamine sulfate. Successfully infected cells were selected using 1 μg/ml puromycin.

### Immunoblotting

For protein lysate generation from cultured cells, cells were washed twice with cold PBS and then manually scraped in the presence of 50-200 μl lysis buffer (25mM Tris-phosphate, pH 7.8; 2mM DTT; 2mM 1,2-diaminocyclohexane-N,N,N′,N′-tetraacetic acid; 10% glycerol; 1% Triton X-100). Cell lysates were incubated on ice for 15 min with 10 sec vortex shaking every five min. Finally, lysates were spun at 10.000 *g* for 2 min at 4°C. Supernatants were recovered and either used immediately or stored for subsequent use. Protein lysates from primary tumors were obtained by mechanically homogenizing the tumors using a mortar and a pestle in cold PBS supplemented with anti-protease and anti-phosphatase cocktails. After homogenization samples were washed twice in cold PBS and the pellets were used for lysate preparation. Protein concentration was determined according to standard Bradford assay. Proteins (35 μg) were separated by SDS-PAGE, transferred to a PVDF membrane (Immobilon-P, Millipore S.p.A., Vimodrone, Italy), and detected with the following antibodish: anti-phospho-AKT (Ser473) (#4058, Cell Signaling Technology, Danvers, MA, USA), anti-AKT (#9272, Cell Signaling Technology), anti-phospho-p44/42 MAPK (ERK1/2) (Thr202/Tyr204) (#4377, Cell Signaling Technology), anti-p44/42 MAPK (Erk1/2) (#9102, Cell Signaling Technology), anti-phospho-Rb (Ser807/811) (#9308, Cell Signaling Technology), anti-Rb (#9309, Cell Signaling Technology), anti-HRAS (Santa Cruz Biotechnology) and anti-β-actin (Santa Cruz Biotechnology). Band intensities were quantified by Quantity One SW software (Bio-Rad Laboratories, Inc) using standard ECL.

### Gene expression microarray and functional analyses

For total RNA isolation, cells were lysed in RLT buffer and RNA was isolated with the RNeasy Mini Kit according to the manufacturer's instructions (Quiagen, GmbH Hilden, Germany). RNAs were hybridized in quadruplicate on Agilent Human GE 4x44K V2 Microarray (G2519F-026652) following the manufacturer's protocol. Hybridized microarray slides were scanned with the Agilent DNA Microarray Scanner G2505C at a 5 μm resolution with the manufacturer's software (Agilent ScanControl 8.1.3). The scanned TIFF images were analyzed numerically and background-corrected using the Agilent Feature Extraction Software (version 10.7.7.1), according to the Agilent GE1_107_Sep09 standard protocol. The output of Feature Extraction was analyzed with the R software environment for statistical computing (http://www.r-project.org/) and the Bioconductor library of biostatistical packages (http://www.bioconductor.org/). Low signal Agilent probes, identified by a repeated “not detected” flag across the majority of the arrays in every condition, were filtered out from the analysis. Signal intensities across arrays were normalized with the quantile normalization algorithm. DEGs were determined adopting a double threshold based on 1) the magnitude of the change (log2 fold change >1 and <-1 for induced and repressed genes, respectively); 2) the statistical significance of the change, measured with a moderated t-test (p-value <0.05) implemented in the Bioconductor Limma package. The DAVID resource was used for enrichment analysis of the DEGs lists, using annotations from Gene Ontology (http://www.thegeneontology.org), KEGG (http://www.genome.jp/kegg/), and PFAM (http://pfam.sanger.ac.uk/). The significance of gene lists over-representation was determined using a p-value threshold of 0.05. The Ingenuity Pathway Analyses (IPA) resource was also used for enrichment analysis of the transcriptome DEGs lists. The IPA Ontologies are derived from manually curated collections of experimental data and are utilized to infer functional enrichment of different type of relationships. The significance of gene list over-representations was determined using a Fisher exact p-value threshold of 0.05. All microarray data are available through the Gene Expression Omnibus database (http://www.ncbi.nlm.nih.gov/geo/) using the accession number GSE62663.

### Quantitative real time PCR (QPCR)

Total RNA was extracted from cells using RNeasy mini kit (Qiagen S.r.l., Milan, Italy) according to the manufacturer's instructions. 1 μg RNA was reverse transcribed in a final volume of 50 μl using High Capacity cDNA Reverse Transcription kit (Life Technologies, Monza, Italy). 5 μl of the resulting cDNA were used for qPCR with a 7900 HT Fast Real-Time PCR (Applied Biosystems by Life Technologies, Monza, Italy). Primer sequences were as follows: E2F1: FW 5′- ATGTTTTCCTGTGCCCTGAG -3′; REV 5′- ATCTGTGGTGAGGGATGAGG -3′; E2F2: FW 5′- CTCTCTGAGCTTCAAGCACCTG -3′; REV 5′- CTTGACGGCAATCACTGTCTGC -3′; RAD51: FW 5′- TCTCTGGCAGTGATGTCCTGGA -3′; REV 5′- TAAAGGGCGGTGGCACTGTCTA -3′. mRNA levels were detected using SYBR Green GoTaq® qPCR Master Mix (Promega Italia S.r.l., Milan, Italy) according to the manufacturer's protocol. Gene expression was normalized to housekeeping gene expression (β-Actin). Comparisons in gene expression were calculated using the 2^−ΔΔCt^ method.

### Animal experiments

All mouse experiments were performed in accordance with the relevant laws and institutional guidelines for animal care and use following approval by the Institutional Animal Care and Use Committee of the Advanced Biotechnology Center (Genoa, Italy; protocol #355). Six to eight week old female BALB/c athymic (nu^+^/nu^+^, n=24) mice were acquired from Charles Rivers Laboratories (Paris, France). Mice were housed in air-filtered laminar flow cabinets with a 12 -hour light cycle and food and water ad libitum. Mice were acclimatized for 2 weeks. 5 × 10^6^ H3122 cells or 2 × 10^6^ HCT116 cells were injected subcutaneously. Treatment was initiated when the tumors appeared as established palpable masses (~2 weeks after the injection). In each experiment, mice were randomly assigned to one of four arms (with 6-8 mice per treatment arm): control - normal diet; TKI -normal diet with 3 cycles of TKI (25 mg/kg/day crizotinib in 0.5% hydroxypropylmethycellulose, 0.1 % Tween 80; or 7 mg/kg/day regorafenib 30% 1,2 Propylenglykol, 30% PEG400, 10% Kolliphor 188, via oral gavage for 5 days a week, Mon-Fri); fasting [water only, for 48h (Sun-Tue) for three cycles at 1-week intervals]; fasting+TKI. Body weight and tumor volume were recorded daily. Tumor volume was calculated using the formula: tumor volume= (w2 × W) x π/6, where “w” and “W” are “minor side” and “major side” (in mm), respectively. Mice were sacrificed after three weeks of treatment. Tumor masses were isolated, weighted, and used for protein lysate preparation and subsequent immunoblotting experiments.

### Statistical analyses

All data are represented as mean ± SD of at least three independent experiments. Statistical analyses were performed with GraphPad Prism software version 5 (GraphPad Software) using one-way ANOVA for multiple-group comparison or unpaired t-test for two-group comparison. p values below 0.05 were considered significant. The cooperative index (CI) was calculated as the sum of the specific cell deaths induced by the single agents divided by the specific cell death in response to the combination. CI values <1, = 1 and >1 indicate a synergistic, additive or infra-additive effect respectively [28].

## SUPPLEMENTARY MATERIALS, FIGURES, TABLES










