# Supplementary material for: Fasting potentiates the anticancer activity of tyrosine kinase inhibitors by strengthening MAPK signaling inhibition
Source: Oncotarget. 2015 Mar 18;6(14):11820–32. doi: 10.18632/oncotarget.3689 (PMC4494907; doi:10.18632/oncotarget.3689)
Supplement: Supplementary file 1 [file oncotarget-06-11820-s001.pdf]

Fasting potentiates the anticancer activity of tyrosine kinase inhibitors by strengthening MAPK signaling inhibition

Supplementary Material

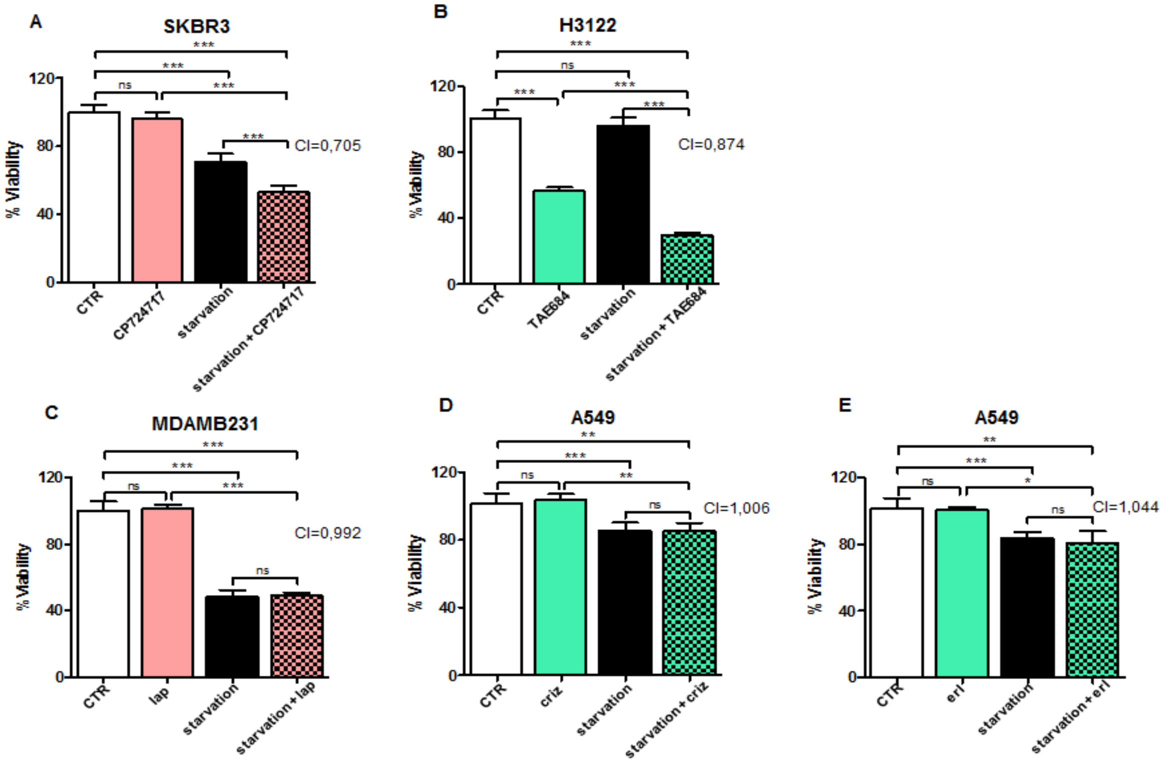

Supplementary Figure 1: Starvation conditions synergistically increase the antiproliferative effects of TKIs in cultured cancer cells without affecting the specificity of these agents. A-E, SKBR3, H3122, MDA-MB-231 (triple-negative, mesenchymal-like breast cancer), and A549 (NSCLC, WT EGFR) cells were plated in 96 well plates in regular culture medium. 24 h later, the cell medium was removed and cells were incubated either in regular medium (CTR) or in starvation medium (starvation). 24 h later, 100 nM of the HER2 TKI CP724717 (SKBR3, A), 100 nM of the ALK TKI TAE784 (H3122, B), 100 nM lapatinib (MDA-MB-231, C), 400 nM crizotinib (criz, A549, D), or 100 nM erlotinib (erl, A549, E) were added where indicated. 72 h later, viability was detected by CellTiter96 Aqueous1. CIs for the combination TKI-starvation are indicated within each panel.

Supplementary Table 1: DEGs among different conditions in H3122 cell treated with starvation, crizotinib, or their combination

#	Condition	p-value<0.001	Up	Down
1	STARVATION vs. CTR	2308	1133	1175
2	CRIZ vs. CTR	1305	666	639
3	STARVATION vs. CRIZ	568	32	536
4	CRIZ+STARVATION vs. CTR	2676	1492	1184
5	CRIZ+STARVATION vs. STARVATION	334	192	142
6	CRIZ+STARVATION vs. CRIZ	264	82	182

Supplementary Table 2: Functional categorization of genes downregulated during crizotinib treatment

Supplementary Table 3: Common Genes in the first top four GO Categories of cells treated with starvation or with crizotinib

Supplementary Table 4: Functional Categorization of DEGs of cells treated with starvation plus crizotinib vs. crizotinib alone

Supplementary Table 5: IPA of gene expression profiles in H3122 cells treated with crizotinib plus starvation vs. crizotinib alone
